# Micronutrient intake status and associated factors in children aged 6–23 months in sub-Saharan Africa

**DOI:** 10.1038/s41598-023-36497-3

**Published:** 2023-06-22

**Authors:** Melaku Tadege Engidaw, Alemayehu Digssie Gebremariam, Sofonyas Abebaw Tiruneh, Desalegn Tesfa, Yalelet Fentaw, Belayneh Kefale, Mulu Tiruneh, Abebaw Tadesse Wubie

**Affiliations:** 1 Department of Public Health (Human Nutrition), College of Health Sciences, Debre Tabor University, P.o. Box: 272, Debre Tabor, Ethiopia; 2 Department of Public Health (Epidemiology), College of Health Sciences, Debre Tabor University, P.o. Box: 272, Debre Tabor, Ethiopia; 3 Department of Public Health (Reproductive Health), College of Health Sciences, Debre Tabor University, P.o. Box: 272, Debre Tabor, Ethiopia; 4 Department of Nutritional Care and Counseling, University of Gondar Specialized Hospital, P.o. Box: 196, Gondar, Ethiopia; 5 Department of Pharmacy (Clinical Pharmacy), College of Medicine and Health Sciences, Bahir Dar University, P.O. Box 79, Bahar Dar, Ethiopia; 6 Department of Public Health (Biostatistics), College of Health Sciences, Debre Tabor University, P.o. Box: 272, Debre Tabor, Ethiopia; 7 Department of Mathematics, College of Natural and Computational Sciences, Debre Tabor University, P.o. Box: 272, Debre Tabor, Ethiopia

**Keywords:** Health care, Medical research, Risk factors

## Abstract

Micronutrient deficiency has serious consequences across all ages worldwide, particularly in sub-Saharan Africa. Poor micronutrient (MN) consumption among children remains a major public health concern in many countries. Low literacy, poor diets, a lack of access to healthcare, and insufficient agricultural productivity made proper micronutrient consumption difficult, despite numerous interventions. Thus, this research aimed to determine the adequate intake of MNs among 6–23 months of age children in sub-Saharan Africa. Initially, a two-stage stratified sampling technique was applied for the selected recent demographic and health survey data. The data source was the (2015–2020) standard demographic and health surveys (DHS) among 20 Sub-Saharan African countries. Micronutrient intake status (the outcome variable) was determined using either food rich in Vitamin A or iron consumed within the previous 24 hr or multiple micronutrient powder or iron supplementation within the previous 07 days or vitamin A supplementation or deworming within 06 months. A generalized linear mixed model based on Modified Poisson regression and robust error variance was used to identify factors associated with children’s adequate micronutrient intake. An adjusted Prevalence Ratio (aPR) with a 95% confidence interval was used to identify factors, strength and direction of the association. The total samples of 65,187 children aged 6–23 months were included. Of all, 73.99% (95% CI: 73.65, 74.32) of children had adequate intake of micronutrients. Maternal education (primary (aPR = 1.04, 95% CI: 1.02, 1.06) and secondary (aPR = 1.07, 95% CI: 1.04, 1.09)), father’s education (primary (aPR = 1.03, 95% CI: 1.01, 1.05) and secondary (aPR = 1.04, 95% CI: 1.02, 1.06)), occupational status of the mother (aPR = 1.04, 95% CI: 1.02, 1.06), pregnancy interval (aPR = 0.97, 95% CI: 0.95, 0.99), exclusive breastfeeding status (aPR = 0.83, 95% CI: 0.82, 0.85), birthweight (average (aPR = 1.03, 95% CI: 1.01, 1.05) and larger than average (aPR = 1.04, 95% CI: 1.02, 1.06)), multiple/twin at birth (aPR = 0.94, 95% CI: 0.91, 0.98), child age (aPR = 1.22, 95% CI: 1.19, 1.25), number of children in home (aPR = 1.02, 95% CI: 1.01, 1.03), ANC utilization (aPR = 1.20, 95% CI: 1.15, 1.27), place of birth (AOR = 0.93, 95% CI: 0.91, 0.95), rich households (aPR = 1.03, 95% CI: 1.01, 1.05), and countries from Central (aPR = 1.07, 95% CI: 1.04, 1.09), South Africa (aPR = 1.07, 95% CI: 1.03, 1.11), and West African (aPR = 0.95, 95% CI: 0.92, 0.99) were associated with level of micronutrients intake status. The prevalence of adequate intake of MN was considerable. Variables at the child, family and community levels were associated with adequate intake of micronutrients. Consequently, stakeholders’ involvement is required in healthcare and community settings.

## Introduction

One in two under-five children suffers from hidden hunger (micronutrient deficiency), which accounts for more than 340 million cases^1^ and is associated with the highest burden of diseases in Africa^2^. As a result, significant numbers of deaths were contributed by both micro and macronutrient deficiencies^3^. So, adequate nutrition during childhood is critical for optimal growth, health and development. Falling to meet the body requirement due to inadequate intake, highest losses and increased demand will lead to growth faltering, micronutrient deficiencies and common childhood illnesses^4,5^. Globally, micronutrient malnutrition is very prevalent^6^ and significantly contributes to child morbidity and mortality^7^. A billion individuals were predicted to be deficient in essential micronutrients (vitamins and minerals) like vitamin A, iodine and zinc^8^. Mostly in Africa, millions of vulnerable groups like children face one form of Micronutrient Deficiency^9^ regardless of different micronutrient deficiency prevention strategy the implementation^10^.

Nowadays, according to our knowledge, there is no database to show adequate intake of micronutrients worldwide including sub-Saharan African (SSA) countries. But there are pocket studies in including sub-Saharan African (SSA) countries to show the magnitude of components of micronutrient intake. In Ethiopia, 37.3% of children aged 6–23 months had not received any recommended micronutrients (MNs) sources^11^. Another systematic review (four countries: Ethiopia, Nigeria, Kenya, and South Africa) found that inadequate intakes varied from 51 to 99% for zinc, 13 to 100% for iron, and 1 to 100% for vitamin A, with households consuming iodized salt at rates ranging from 2 in Kenya to 96% in Ethiopia^12^. In addition, a study in South Africa shows that preschool children and their caregivers consumed a diet deficient in most of the essential micronutrients; due to poor quality of the diet with low vitamin A and iron status, one-fifth of the children had linear growth retardation^13^. Furthermore, a study in the pastoralist community of Kenya suggests that the highest probability of inadequacies for vitamins A, B12, and C and this is associated with different individual and household level factors^14^.

The most common contributors for inadequate micronutrient intakes are difference in residency area and geography^11^, racial differences, lower education and self-reported economic-related food insecurity^15^, monotonous diet, low bioavailability, seasonal variations and eating phytate foods as staple foods^8,16^. On the other hand, lack of availability and access to health care facilities, trained personnel and poor health care set-ups were also a factor that hinder the adequate services^4^.

The problem is escalating in developing countries due to a lack of consumption of the recommended sources of micronutrients like grains, roots and tubers, legumes and nuts, dairy products, flesh foods (meat, fish, poultry, and organ meats), eggs, vitamin A rich fruits and vegetables, and other fruits and vegetables consumed within the previous 24 h^17^. Hence, to address the scarcity of literature on the significant effect of individual and/or community level factors on adequate intake of micronutrients in SSA, this study utilized a multilevel modified Poisson regression model. As a result, this study sought to identify the individual and/or community-level factors associated with adequate micronutrient intake in children aged 6 to 23 months in sub-Saharan African countries.

## Methods

### Data sources

The data source was the recent (2015–2020) standard demographic and health survey (DHS) among 20 sub-Saharan African countries. The data were obtained from the DHS program (http://www.dhsprogram.com/) after a formal written request. A nationally representative standard DHS was used to estimate the pooled magnitude of micronutrient intake status among children 6–23 months of age in sub-Saharan Africa. The data were collected through a multistage stratified cluster sampling technique for each country. The nature of the data is hierarchal since the individuals are nested in regions and nations.

From all SSA countries, 17 countries datasets (namely: Benin, Burundi, Cameroon, Ethiopia, Gambia, Guinea, Liberia, Mali, Malawi, Nigeria, Rwanda, Sierra Leone, Tanzania, Uganda, South Africa, Zambia, and Zimbabwe) were included for this study. All other countries' data sets were excluded due to the absence of dataset, old dataset, and no denominator and numerator variables according to the guide to DHS statistics version 7.2^18^.

### Study population

For this study, all children 6–23 months of age with their mothers’ or caregivers’ records or data were the study population.

### Study variables

In this study, the outcome variable is micronutrient intake status. Micronutrient intake status was determined using either food rich in Vitamin A or iron consumed within the previous 24 hr or multiple micronutrient powder or iron supplementation within the previous 07 days or vitamin A supplementation or deworming within 06 months^18^.

Individual or community level, or both factors, affected the micronutrient intake status of children aged 6 to 23 months.

### Individual-level variables

Both maternal (socio-demographic and maternal health service utilization from pregnancy to postnatal time) and child-related variables have been included at this stage.

### Community-level variables

As community-level variables, the place of residence, economic region, and the country was included.

### Data processing and analysis

After obtaining the data set for each country from DHS programs, the data analysis was explored using Stata 16/MP (Stata Corporation IC., TX, USA) for Windows. The wealth index is obtained directly from the dataset that ranges from poorest to richest. Due to the hierarchical nature of DHS data, the observation of the data violates the assumption of independence. To assess the variation among the cluster, we estimated the Intra-class Correlation Coefficient (ICC) and deviance values. The outcome variable (adequate intake of MNs) was determined according to DHS statistics guide 7.2 using the following variables: foods rich in vitamin A and iron, supplementation of multiple micronutrient powder (MNP), iron, vitamin A and or deworming.

We were forced to use a generalized linear mixed model with Poisson regression (modified) and the robust error of variance to identify factors associated with micronutrient intake in SSA because the outcome variable, MN intake status among children 6–23 months of age, is (73.99%). Then, we have fitted four models: the null model (without independent variables), the model I (only individual-level variables (child and maternal sociodemographic variables)), model II (only community-level variables (residence and region)), and model III (both the individual and community level variables). Model III had the smallest deviance (-2LL) value of all models and was chosen as the best-fitted model for the discussion of this study result.

Before building all these models, a bi-variable generalized linear mixed (GLM) effect model using modified Poisson regression with the robust error of variance was employed to identify candidate variables for multivariable analysis in each category. Finally, a multivariable multilevel GLM effect model using Modified Poisson regression with the robust error of variance analysis was done for variables with a p-value < 0.25 during the bi-variable analysis (for initial assessment/screening). We have calculated the unadjusted and adjusted prevalence ratios (aPR) with 95% CI. We declare the statistical significance of variables and the degree of strength of the effect size by estimating the Confidence Intervals.

### Parameter estimation method

In this study, parameters were estimated as follows:

- Intra-class correlation Coefficient (ICC) was calculated as; ICC = $\frac{\left({\mathrm{\sigma \mu }}^{2}\right)}{\upsigma {\upmu }^{2}+\upsigma {\mathrm{e}}^{2}}$ , where σμ^2^ is the variance of the group level; and σe^2^ denotes the variance of the individual level^19^.
- Proportional change in variance (PCV) was calculated as; PCV $=\frac{\left({V}_{0}-{V}_{x}\right)*100}{{V}_{0}}$, where V_0_ is the variance of the null model and V_x_ is each the variance of each model at each level with variables^19^.
- Prevalence median ratio (PMR) was calculated as; PMR = exp $\sqrt{2{\upsigma }^{2}*{\Phi }^{-1}(3/4)]}$, where σ^2^ is the variance of each model and Φ^−1^ is inverse of the standard normal cumulative distribution function^20^

### Ethical consideration

The International Review Board of Demographic and Health Surveys (DHS) program data archivists were allowed to download and use the datasets for this study. Also, the data was handled according to the Helsinki Declaration of the World Medical Association.

## Results

### Sociodemographic characteristics of the participants

This report included 65,187 children aged 6 to 23 months from each nation (17 SSA countries). The mean ± SD of the children’s age was 12.79 ± 6.43 months. The mean ± SD of the mother’s/caregivers age was 27.96 ± 6.82 years. Of all, 70.96% of the mothers/caregivers found between 20 and 34 years of age. More than one-third and one-fourth of the mothers in sub-Saharan Africa have not attended any formal education and no formal work, correspondingly (Table 1).

**Table 1:** Sociodemographic characteristics of study participants in sub-Saharan Africa, 2015–2020 (n = 65,187).

Variables		Frequency	Percent (%)
Mother’s age (in completed years)	< 20 years	6183	9.49
	20 – 34 years	46,258	70.96
	35 – 49 years	12,746	19.55
Marital status	Not married	4746	7.28
	Married	60,441	92.72
Maternal educational status	No formal education	23,509	36.06
	Primary education	22,418	34.39
	Secondary education	19,260	29.55
Father educational status**	No formal education	18,750	33.76
	Primary education	17,195	30.96
	Secondary and above education	19,589	35.27
Head of the household	Male	52,675	80.81
	Female	12,512	19.19
Maternal occupation status**	Not working	16,530	26.88
	Working	44,976	73.12
Father occupation status**	Not working	3195	5.70
	Working	52,830	94.30
Family size	≤ 5	28,357	43.50
	> 5	28,357	56.50
Child’s age	6 – 11 months	23,223	35.63
	12 – 23 months	41,964	64.37
Child’s sex	Male	31,975	50.95
	Female	33,212	49.05
Residence	Urban	18,691	28.67
	Rural	46,496	71.33
Country	Benin	4,781 7.33	7.33
	Burundi	4604	7.06
	Cameroon	3275	5.02
	Ethiopia	3472	5.33
	Gambia	2855	4.38
	Guinea	2523	3.87
	Liberia	1943	2.98
	Mali	3372	5.17
	Malawi	5697	8.74
	Nigeria	11,050	16.95
	Rwanda	2706	4.15
	Sierra Leone	3389	5.20
	Tanzania	3725	5.71
	Uganda	5244	8.04
	South Africa	1158	1.78
	Zambia	3382	5.19
	Zimbabwe	2011	3.08
Country income	Poor	30,279	46.45
	Middle	12,912	19.81
	Rich	21,996	33.74
Economic regions	East Africa	30,841	47.31
	Central Africa	3275	5.02
	South Africa	1.158	1.78
	West Africa	29,913	45.89

**missing value.

### Reproductive health-related characteristics

Of all, 44,667 (68.52%) respondents were < 20 years of age at their first pregnancy. According to this pooled data, the magnitude of institutional delivery for the index child was 46,628 (71.53%). Only one-third of the respondents were starting ANC follow-up before the end of the first trimester. One fourth of the mothers did not exclusively breastfeed their children (Table 2).

**Table 2:** Reproductive health characteristics of study participants and respondents in Sub-Saharan Africa, 2015–2020 (65,187).

Variables		Frequency	Percent (%)
Age at 1st pregnancy	< 20 years	44,667	68.52
	20 – 49 years	20,520	31.48
Birthplace	Health institution	46,628	71.53
	Home	18,559	28.47
Mode of delivery**	Spontaneous vaginal delivery	61,334	94.30
	Cesarean section	3710	5.70
Antenatal care follow-up status	No antenatal care follow up	6237	9.57
	Have antenatal care follow up	55,372	84.94
	Don't know	3578	5.49
Time to start ANC* follow up	< 12 weeks	22,004	33.76
	≥ 12 weeks |	43,183	66.24
plurality of birth	Single	62,837	96.39
	Multiple	2350	3.61
Birth weight**	Smaller than average	10,749	16.74
	Average	32,940	51.29
	Larger than average	20,539	31.98
Exclusive breastfeeding status	Yes	48,024	73.67
	No	17,163	26.33
Preceding birth interval**	≥ 24 months	42,212	83.83
	< 23 months	8140	16.17
Pregnancy status at the time of interview	No or unsure	61,450	94.27
	Yes	3737	5.73
Media exposure	Yes	59,628	91.47
	No	5559	8.53

*ANC antenatal care. **missing value

### Cluster variation analysis

The measure of variation was measured for each model through Intra Class Correlation (ICC), Median Prevalence Ratio (MPR), and deviance. In the null model, the value of ICC was 10.67% (95% CI: 8.68, 13.06), which shows the presence of heterogeneity of micronutrient intake status among regions (clusters), as also indicated by the MPR with a value of 1.11. It means that, if we randomly select a child, a child in the regions with higher micronutrient intake status was 1.11 times higher as compared to a child in regions with lower micronutrient intake status.

Of all models that measure variations, model III has the lowest MPR and the deviance value, which was 1.04 and 34,163.07, respectively. So, model III was selected as the best-fitted model for this analysis (Table 3).

**Table 3:** Variation in the fitness of each model for micronutrient intake status among children aged 6 to 23 months in sub-Saharan Africa, 2015–2020.

Parameters	Model I^a^	Model II^b^	Model III^c^	Model IV^d^
ICC (%)	10.67 (8.68, 13.06)			
Community level variance (95% CI)	0.012 (0.007, 0.020)	0.003 (0.001, 0.006)	0.009 (0.005, 0.017)	0.003 (0.001, 0.005)
PCV	Reference	74.08	19.93	79.01
MPR (95% CI)	1.11 (1.10, 1.12)	1.05 (1.04, 1.06)	1.09 (1.08, 1.11)	1.05 (1.05, 1.06)
LL (AIC)	− 56,889.55 (113,783.1)	− 34,172.59 (68,395.18)	− 56,858.87 (113,733.7)	− 34,163.07 (68,388.14)
Deviance (− LL)	113,779.1	68,345.18	113,717.74	68,326.14

*CI* confidence interval, *ICC* inter class correlation, *LL* log likelihood, *MPR* median prevalence ratio, *PCV* proportional change in variance.

^**a**^null model, ^**b**^Adjusted for individual-level variables, ^**c**^Adjusted for community level variables, ^**d**^Full/final model (adjusted for individual and community-level variable).

### The pooled magnitude of micronutrient intake status in sub-Saharan Africa

The overall magnitude of adequate micronutrient intake status among children 6 – 23 months of age in sub-Saharan Africa was (73.99%, 95% CI 73.65, 74.32). The highest micronutrient intake status was found in Burundi (85.53%) and Rwanda (85.48%) followed by Malawi (82.18%) while the lowest was found in Ethiopia (59.10%) followed by Guinea (60.05%) as shown in the table below (Table 4). In sub-Saharan Africa, the proportion of recommended MNs consuming Vitamin A rich foods, Iron-rich foods, MMPs, Iron supplementation, Vitamin A supplementation, and deworming was (35,934 (55.12%), (22,568 (34.62%)), (2247 (3.45%)), (8670 (13.30%)), (33,497 (51.39%)), and (20,939 (32.12%)), respectively.

**Table 4:** Micronutrient intake status among children aged 6 to 23 months in each sub-Saharan African country, 2015–2020 (n = 65,187).

Countries	Year of survey	Total number of children	Frequency adequate micronutrient intake	Proportions of adequate micronutrient intake
Benin	2017/18	4781	3477	72.73
Burundi	2016/17	4604	3938	85.53
Cameroon	2018/19	3275	2524	77.07
Ethiopia	2016	3472	2052	59.10
Gambia	2019/20	2855	2196	76.92
Guinea	2018	2523	1515	60.05
Liberia	2019/20	1943	1409	72.52
Mali	2018	3372	2475	73.40
Malawi	2015/16	5697	4682	82.18
Nigeria	2018	11,050	7015	63.48
Rwanda	2019/20	2706	2313	85.48
Sierra Leone	2019	3389	2597	76.63
Tanzania	2015/16	3725	2728	73.23
Uganda	2016	5244	4130	78.76
South Africa	2016	1158	928	80.14
Zambia	2018/19	3382	2649	78.33
Zimbabwe	2015	2011	1604	79.76
Total		65,187	48,232	73.99

### Factors associated with micronutrient intake status

Initially, a bi-variable multilevel GLM model using Poisson regression with robust error variance was used to identify factors associated with adequate micronutrient intake status of children 06 – 23 months of age in sub-Saharan African Countries. A separate model for individual, community, or both (community and individual level) factors were employed. From all models, the fourth model, which contained both individual and community level factors were identified as the best fit model with the lowest negative likelihood ratios (− 34,163.07(AIC = 68,388.14)) or deviance (68,326.14) values.

During bi-variable analysis; Age of the respondent, maternal educational status, father's educational status, maternal occupational status, father's occupational status, family size, age at first pregnancy, pregnancy interval, exclusive breastfeeding status, the weight of the child at birth, plurality, age of the child, mode of delivery, number of children, ANC Utilization, time to start ANC follow up, place of delivery, media exposure, residence, wealth Index, and economic regions of SSA countries were the significant variables.

All these variables were fitted into a multivariable multilevel GLM model using Poisson regression with the robust error of variance analysis. Finally, maternal educational status, father's educational status, maternal occupational status, pregnancy interval, exclusive breastfeeding, the weight of the child at birth, plurality, age of the child, number of children, ANC Utilization, time to start ANC follow-up, place of delivery, wealth index, and economic regions of SSA countries were the associated factors with adequate intake of micronutrients (MNs) status among 06 – 23 months of age children (Table 5).

**Table 5:** Multivariable multilevel modified Poisson regression analysis of factors associated with micronutrient intake status in children aged 06 – 23 months in sub-Saharan Africa, 2015 – 2020.

Variables		Model I^a^ (aPR, 95% CI)	Model II^b^ (aPR, 95% CI)	Model III^c^ (aPR, 95% CI)	Model IV^d^ (aPR, 95% CI)
Age of the respondent	< 20 years		1		1
	20–34 years		1.05 (0.99, 1.10)		1.05 (0.99, 1.11)
	35–49 Years		1.03 (0.98, 1.09)		1.03 (0.98, 1.09)
Maternal educational status	No formal education		1		1
	Primary education		1.05 (1.03, 1.07)		1.04 (1.02, 1.06)
	Secondary education		1.08 (1.06, 1.10)		1.07 (1.04, 1.09)
Father’s educational status	No formal education		1		1
	Primary education		1.04 (1.02, 1.06)		1.03 (1.01, 1.05)
	Secondary education and above		1.05 (1.03, 1.07)		1.04 (1.02, 1.06)
Maternal occupational status	Not working		1		1
	Working		1.03 (1.01, 1.05)		1.04 (1.02, 1.06)
Father’s occupational status	Not working		1		1
	Working		1.01 (0.98, 1.05)		1.01 (0.98, 1.04)
Family size	≤ 5		1		1
	> 5		1.01 (0.98, 1.02)		1.01 (0.99, 1.02)
Age at first pregnancy	Not teen		1		1
	Teen		1.01 (0.97, 1.03)		1.01 (0.97, 1.03)
Pregnancy interval	≥ 24 months		1		1
	< 23 months		0.97 (0.95, 0.99)		0.97 (0.95, 0.99)
Exclusive breastfeeding	Yes		1		1
	No		0.84 (0.82, 0.86)		0.83 (0.82, 0.85)
Weight of the child at birth	Smaller than average		1		1
	Average		1.03 (1.01, 1.05)		1.03 (1.01, 1.05)
	Larger than average		1.04 (1.01, 1.06)		1.04 (1.02, 1.06)
Plurality	Single		1		1
	Multiple		0.94 (0.91, 0.94)		0.94 (0.91, 0.98)
Age of the child	6 – 11 months		1		1
	12 – 23 months		1.22(1.19, 1.25)		1.22 (1.19, 1.25)
Mode of delivery	SVD **		1		1
	Cesarean section		0.98 (0.97, 1.01)		0.98 (0.96, 1.01)
Number of children	< 4		1		1
	≥ 4		1.02 (1.01, 1.03)		1.02 (1.01, 1.03)
ANC* utilization	Yes		1.20 (1.15, 1.26)		1.20 (1.15, 1.27)
	No		1		**1**
	Don’t know		1.15 (1.07, 1.24)		1.16 (1.08, 1.25)
Time to start ANC* follow up	< 12 weeks		1		1
	≥ 12 weeks		0.99(0.98, 1.01)		0.99 (0.98, 1.01)
Place of delivery	Health institution		1		1
	Home		0.93 (0.91, 0.95)		0.93 (0.91, 0.95)
Media exposure	Yes		1.02 (1.01, 1.03)		1.01 (0.99, 1.02)
	No		1		1
Residence	Rural			0.98 (0.97, 1.01)	0.98 (0.97, 1.01)
	Urban			1	1
Wealth Index	Poor			1	1
	Middle			1.03 (1.02, 1.05)	1.01 (0.98, 1.02)
	Rich			1.06 (1.04, 1.09)	1.03 (1.01, 1.05)
Economic regions of SSA countries	East Africa			1	1
	Central Africa			1.01 (0.96, 1.04)	1.07 (1.04, 1.09)
	South Africa			1.02 (0.99, 1.05)	1.07 (1.03, 1.11)
	West Africa			0.91 (0.88, 0.95)	0.95 (0.92, 0.99)

1 = Reference, *CI* confidence interval, *ANC* antenatal care, *aPR* adjusted prevalence ratio, **SVD spontaneous vaginal delivery, ^**a**^null model, ^**b**^adjusted for individual-level variables, ^**c**^adjusted for community level variables, ^**d**^full/final model (adjusted for individual and community-level variable).

Children from a mother with primary and secondary education were 4% (aPR = 1.04, 95% CI: 1.02, 1.06) and 7% (aPR = 1.07, 95% CI: 1.04, 1.09) higher prevalence of adequate intake of MNs as compared to those who were from mothers without any formal education. Children from a father with primary and secondary education were 3% (aPR = 1.03, 95% CI: 1.01, 1.05) and 1.04 (aPR = 1.04, 95% CI: 1.02, 1.06) more prevalence of adequate intake of MNs as compared to those who were from a father without any formal education.

Children who were from a mother with any job had a 4% (aPR = 1.04, 95% CI: 1.02, 1.06) higher prevalence of adequate intake of MNs as compared to their counterparts. Also, children born with a pregnancy interval of fewer than 23 months were a 3% (aPR = 0.97, 95% CI: 0.95, 0.99) lower prevalence of adequate intake of MNs as compared to their counterparts. Also, no exclusive breastfeeding children had a 17% (aPR = 0.83, 95% CI: 0.82, 0.85) lower prevalence of adequate intake of MNs as compared to those who have exclusive breastfeeding.

Similarly, children born with average and larger than average were 3% (aPR = 1.03, 95% CI: 1.01, 1.05) and 4% (aPR = 1.04, 95% CI: 1.02, 1.06) higher prevalence of adequate MNs as compared to children born as smaller than average. Also, children born multiple/twin were had 6% (aPR = 0.94, 95% CI: 0.91, 0.98) lower prevalence of adequate intake of MNs as compared to children born single.

Children 12 to 23 months of age were a 22% (aPR = 1.22, 95% CI: 1.19, 1.25) higher prevalence of adequate intake of MNIS as compared to children 6 to 11 months of age. An index child with more than 4 children at home have 2% (aPR = 1.02, 95% CI: 1.01, 1.03) higher prevalence of adequate intake of MNs as compared to children from less than four. The prevalence of having adequate MNs were 20% (aPR = 1.20, 95% CI: 1.15, 1.27) higher than children born from mothers without ANC utilization.

Children born at home were a 7% (AOR = 0.93, 95% CI: 0.91, 0.95) lower prevalence of having an adequate intake of MNs as compared to children born in health institutions. Also, children from rich households were a 3% (aPR = 1.03, 95% CI: 1.01, 1.05) higher prevalence of adequate intake of MNs as compared to children born in poor households.

Besides these, children born from the economic regions of Central and South Africa were a 7% (aPR = 1.07, 95% CI: 1.04, 1.09) and 7% (aPR = 1.07, 95% CI: 1.03, 1.11) higher prevalence of adequate intake of MNs as compared to East African countries respectively. On the other hand, children from West African countries were a 5% (aPR = 0.95, 95% CI: 0.92, 0.99) lower prevalence of adequate intake of MNs as compared to East African countries.

## Discussion

Adequate intakes of micronutrients have a significant role in the optimal health, growth and development of a child. But there is no pooled data related to this and its contributing factors in SSA. Therefore, this study helps to fill this gap by generating evidence among children 6 – 23 months of age.

The overall magnitude of adequate micronutrient intake status among children 6 – 23 months of age in sub-Saharan Africa was (73.99%, 95% CI: 73.65, 74.32) that ranges from 59.1% in Ethiopia to 85% in Burundi and Rwanda. This finding is higher than a study conducted in Ethiopia (63.7%)^11^, South Africa (< 50% of the RDA)^13^ and Bangladesh^21^. Similarly, different studies elsewhere using one of the components to measure micronutrient intake status shows that the intake is less than the recommended shows due to low intake of animal food sources, and the highest tubers and cereals consumption in Africa^22,23^. The possible justification might be variation across regions; the dietary intake is varying across regions due to cultural, availability, accessibility and religious differences. Also, the type of staple food varies in each geographic region as per their beliefs, traditions, food insecurity and literacy.

The intake of adequate micronutrients by the children was more likely among households who had parents (mothers and fathers) with the educational status primary and secondary, while we compared the children from parents/caregivers without any formal education. This finding is in agreement with other studies^22–25^. The higher odds of adequate consumption of micronutrients in children from educated parents might be due to better exposure to media, change in knowledge and behaviors, and increasing purchasing power of different food items.

Likewise, children who had a mother with any job had a higher prevalence of adequate intake of MNs as compared to their counterparts which is in line with other different studies^3,26,27^, the possible justification might be the strong association between job and income; a mother with regular income may have purchasing capacity of varieties of food regularly and may be linked with literacy level and exposure to media, which is increasing access to information about feeding practices and consumption of adequate intake of micronutrients.

Also, children who had a pregnancy interval of less than 23 months, born at home and without exclusive breastfeeding had a low level of adequate intake of MNs as compared to their counterparts. Likewise, different studies show that children from mothers/caregivers who were adhering to reproductive health services/issues were more likely to have an adequate intake of MNs, dietary diversity and meal frequency as compared to their counterparts^11,22,26,28^. It is a known fact that women with adequate follow-up will know appropriate infant and child feeding practices due to repeated counselling, which intern helps to improve frequent and diversified foods consumption.

Similarly, children born with average and larger than average were 3% (aPR = 1.03, 95% CI: 1.01, 1.05) and 4% (aPR = 1.04, 95% CI: 1.02, 1.06) higher prevalence of adequate MNs as compared to children born as smaller than average, which is in line with the study conducted in South Africa^13^. Because children's linear growth will be reflected by maternal inadequate intake of nutrients from preconception to date due to lack of variety of foods. This indicates that inadequate nutrients that affect the linear growths were persisting as a problem.

The other socio-demographic factors associated with a lower level of adequate intake of MNs are children who are twins and have more than 4 (four) children at home. Similarly, studies have shown that family size influences children’s dietary intake^29–31^. The possible justification might be the significant associations between food groups consumed. As the family size increase, the portion/meal size, nutrient density, quality of diet and affect the purchasing power of the households, which may affect the level of adequate intake of nutrients. Usually, what is observed is that a greater number of children in the house increases the risk of less adequacy of micronutrients intake.

Like a study in LMICs^32^ and East Africa^23^, this study, children from rich households had an adequate intake of MNs as compared to children born in poor households. This might be due to the purchasing power of households will be associated with overall healthier dietary patterns. Individuals with the highest socioeconomic status (SES) are more likely to consume healthy foods (rich in micronutrients) but not for children belong from low SES.

Children 12 to 23 months of age were a 22% (aPR = 1.22, 95% CI: 1.19, 1.25) higher magnitude of adequate intake of MNIS as compared to children 6 to 11 months of age, which is revealed by the studies conducted East Africa^23^ and Ethiopia^33^. This might be due to the late introduction of complementary feeding, caregivers' perception towards feeding a variety of food for age and beliefs, taboos and practices.

The prevalence of having adequate MNs were 20% (aPR = 1.20, 95% CI: 1.15, 1.27) higher than children born from mothers without ANC utilization. Likewise, a pooled study of young child feeding practices in the East African Region^23^ and iron-rich food consumption^27^ shows the link between maternal health care follow-up and adequate intake of nutrients. The possibility of receiving health information on child nutrition from health care providers during ANC and PNC services may help to change their behaviours and increase appropriate feeding practices, which is a clear indication to promote and offer universal ANC and PNC for mothers in SSA countries to improve micronutrient consumptions.

Besides these, children born from the economic regions of Central and South Africa were more adequate intake level MNs as compared to East African countries, likewise, children from West African countries had a lower level of adequate intake of MNs. Like this, a systematic review reveals that adequate intake of MNs varies across the region including developed countries and Nepal^24^ similarly. This systematic review shows that the existence of shows limited intake of micronutrients in Central and Eastern Europe than in other European countries^25,34^. The association between geographical region/location and adequate intake of MNs will be seasonal variability and type of food sources. Also, it might be due to the variations in education, occupation and income levels across regions. Furthermore, different socioeconomic positions within these countries may exist in greater social inequalities, diversity in food costs, access and availability.

This study's strength is that it attempts to pool adequate micronutrient intake in children aged 6–23 months at the SSA level using a large representative sample. As a result, it is generalizable. It also assessed the impact of individual and community-level variables on MN intake using reliable methods. Furthermore, the study is limited by the cross-sectional nature of the survey, affected recall bias during the interview, time between the survey and deworming medication intake, deworming was used in the last 6 months as an indirect indicator of the adequacy of micronutrients in the diet, a lack of bioavailability data, and an inability to determine the quantity and quality of dietary sources.

## Conclusion

The overall magnitude of adequate intake of micronutrients in the sub-Saharan region among children 06 – 23 months of age is high. Both individual (maternal educational status, father's educational status, maternal occupational status, pregnancy interval, exclusive breastfeeding, weight of the child at birth, plurality, age of the child, number of children, ANC Utilization, time to start ANC follow-up, place of delivery) and community-level (wealth index, and economic regions of SSA countries) variables were associated with the adequate intake of micronutrients (Supplementary Information).

To strengthen further, health care workers at health institutions and community settings shall give attention to promoting means of adequate micronutrient intake. Also, governments shall have to follow the progress activities and strategies related to adequate intake of micronutrients by children routinely.

## Supplementary Information

Supplementary Information.

## Data Availability

The compiled dataset is available from the authors upon reasonable request from corresponding author and with permission of The DHS Program.

## Abbreviations

ANC: Antenatal care
aPR: Adjusted prevalence ratio
CI: Confidence interval
DHS: Demographic and health survey
ICC: Intra-class correlation coefficient
LLR: Log-likelihood ratio
MNs: Micronutrients
MPR: Median odds ratio
PCV: Proportion of coefficient of variations
SSA: sub-Saharan Africa
SVD: Spontaneous vaginal delivery
WHO: World health organization
